# Characteristics and survival in bone metastatic breast cancer patients with different hormone receptor status: A population-based cohort study

**DOI:** 10.3389/fonc.2022.977226

**Published:** 2022-08-26

**Authors:** Xiaofan Jiang, Guanglei Chen, Lisha Sun, Chao Liu, Yu Zhang, Mingxin Liu, Caigang Liu

**Affiliations:** ^1^ Department of Oncology, Shengjing Hospital of China Medical University, Shenyang, China; ^2^ Innovative Cancer Drug Research and Development Engineering Center of Liaoning Province, Shengjing Hospital of China Medical University, Shenyang, China; ^3^ Department of Gastrointestinal Surgery, Yantai Affiliated Hospital of Binzhou Medical University, Yantai, China

**Keywords:** breast cancer, bone metastasis, hormone receptor (HR), HER2 status, SEER (Surveillance Epidemiology and End Results) database

## Abstract

**Background:**

Accumulating preclinical evidence has uncovered the indispensable role of steroid hormone and their receptors, namely, estrogen receptor (ER) and progesterone receptor (PR), in the development of bone metastases in breast cancer. Limited data are available regarding the survival difference between different hormone receptor (HR) subgroups, and its prognostic significance is uncertain now. Such data are important for risk stratification and needed to formulate specialized regimen for bone metastatic breast cancer.

**Methods:**

From the year of diagnosis 2010 to 2018, 554,585 breast cancer patients, among which are 19,439 with bone metastasis and 10,447 with bone-only metastasis, were extracted from the Surveillance, Epidemiology, and End Results (SEER) database. Kaplan–Meier survival analysis was performed to compare the survival difference between the different HR status subgroups. Univariate and multivariate Cox proportional hazard regression was used to validate the prognostic role of HR status and identify other prognostic factors in bone metastatic breast cancer.

**Results:**

ER-positive/PR-positive breast cancer patients with bone metastasis showed the best breast cancer-specific survival (BCSS) and overall survival (OS) than those with other HR statuses, while single PR-positive bone metastatic breast cancers manifest similar survival with ER-negative/PR-negative ones. Adjusted Cox regression analysis demonstrated that patients with older age, male, black race, ILC, higher tumor grade, T3–T4, HER2-negative status, absence of surgery or adjuvant treatment, and HR status other than ER-positive/PR-positive tended to have worse outcomes. Further subgroup analysis based on HER2 status showed that within HER2-positive breast cancers, ER-positive/PR-positive ones still manifest better survival than the other three HR status subgroups, which are similar in survival outcomes.

**Conclusion:**

Although collectively viewed as HR-positive breast cancers, certain distinctions exist between bone metastatic breast cancers with different HR statuses in survival outcome. Our findings indicate that despite metastasizing to the same location, the different survival rate is determined by the HR status of breast cancer. The selection and intensity of the regimen should consider HR status, and HER2 status occasionally, when treating bone metastatic breast cancer.

## Introduction

Despite that the gradually completed early screening and comprehensive treatment strategies have improved the prognosis of breast cancer patients considerably, the occurrence of distant metastasis remains the major cause of cancer-related lethal events (1, 2). Bone is the most common site of metastasis in breast cancer, accounting for 60%–80% of metastatic breast cancer (3, 4), and the median survival for bone metastatic breast cancer ranges from 3 to 5 years (5). Destruction of normal skeletal structure and function after breast cancer cell colonization would cause skeletal-related events (SREs), including severe bone pain, pathological bone fracture, hypercalcemia, and spinal cord compression, leading to reduced quality of life and overall survival (6).

Bone metastatic disease is common across all breast cancer subtypes, while patients with hormone receptor (HR)-positive breast cancer tend to have the greatest predilection for developing bone metastases (7, 8), unlike organotropic metastasis to visceral organs in HER2-positive and triple-negative breast cancer (TNBC) (9, 10). Also, estrogen receptor (ER) status is highly concordant between primary and metastatic bone foci (11, 12), indicating the indispensable role of ER in promoting bone metastasis. Mechanistically, physiological bone metabolism is a well-balanced process maintained by osteoblasts and osteoclasts under regulation of steroid sex hormones and various cytokines (13); a disrupted hormone level would lead to abnormal bone composition. Taken together, sex hormones including estrogen and progestin and their corresponding downstream receptor signaling may be significant for breast cancer bone metastasis formation. Estrogen receptor and progesterone receptor (PR) are both critical steroid HRs routinely employed to predict endocrine therapy response and prognosis in breast cancer (14–16); thus, the HR status of breast cancer could be further subdivided into ER-positive/PR-positive, ER-positive/PR-negative, ER-negative/PR-positive, and ER-negative/PR-negative subpopulations theoretically.

However, studies to date have not comprehensively investigated the demographic characteristics, clinicopathological characteristics, and survival outcomes among bone metastatic breast cancers with these four varied HR statuses. In this retrospective study, we compared the features and survival of these bone metastatic breast cancer subgroups based on the population from the Surveillance, Epidemiology, and End Results (SEER) database. We also validated that HR status is the significant prognostic factor which determines the survival of bone metastatic breast cancers. Collectively, our findings demonstrated that different ER and PR statuses contribute to varied survival outcomes, which may call for taking HR status into consideration and formulating specific strategies while treating bone metastasis in breast cancer.

## Methods

### Cohort data source

The study cohort data were obtained from the SEER database that were released in April 2022 with the SEER*Stat 4.2.0 software (National Institutes of Health, Bethesda, MD). The data include demographic, clinicopathological, treatment, and survival information which cover approximately 34.6% of the US population. Before initiation of this study, we had submitted a data use agreement to the SEER program which was granted with the authorized access (User ID: 10181-Nov2021). Written informed consent from patients was waived because of the public nature of the SEER database. This study was conducted following the Strengthening the Reporting of Observational Studies in Epidemiology (STROBE) reporting guidelines.

### Data collection

Since the bone metastasis status record was available from 2010, the current study extracted the data from the SEER database between 2010 and 2018. A total of 554,585 breast cancer patients with known bone metastasis event and HR status information were included, among which 19,439 patients were diagnosed with bone metastasis. To exclude the potential influence by other malignant comorbidities on survival, patients with metastases to other organs, including brain, lung, and liver, were excluded, leaving 10,447 patients included in survival analysis.

The demographic features, including age at diagnosis, sex, and race, were extracted from the SEER database, as well as the clinicopathological features and survival information, including tumor grade, T staging, N staging, histological type, ER status, PR status, HER2 status, surgery status, chemotherapy status, radiation therapy status, survival months, vital status, and cause of death. The HR status of the included bone metastatic breast cancer was grouped into ER-positive/PR-positive, ER-positive/PR-negative, ER-negative/PR-positive, and ER-negative/PR-negative subgroups. The patients were stratified into the age subgroups of less than 49, 50–69, and more than 70 years, which correspond to young, middle-age, and senior patients, respectively (17). Race was categorized into the white, black, other, or unknown subgroup. Tumor grade was classified as well differentiated (grade I), moderately differentiated (grade II), poorly differentiated (grade III), undifferentiated (grade IV), or unknown subgroup. Histology type was divided into invasive ductal carcinoma (IDC), invasive lobular carcinoma (ILC), collectively as mixed IDC and ILC, or other subgroups. The surgery status was recorded as yes or no/unknown following the SEER Program Coding and Staging Manual 2018; chemotherapy and radiation therapy statuses were recorded as yes or no/unknown.

### Statistical analysis

In this study, all statistical analysis was performed with R software 4.2.0. P values were two-sided, and P < 0.05 was considered statistically significant. The demographic and baseline clinicopathological characteristics of bone metastatic breast cancer patients with ER-positive/PR-positive, ER-positive/PR-negative, ER-negative/PR-positive, and ER-negative/PR-negative primary tumors were analyzed using the chi-square or Fisher’s exact test when appropriate, Bonferroni correction was performed and the significance level was adjusted to 0.008 when comparing the differences in rates or constituent ratios between the four HR subgroups, and P< 0.008 was considered statistically significant. The BCSS was defined as the time from diagnosis to death from breast cancer, and the OS was defined as the time from diagnosis to death from any causes. Kaplan–Meier survival curves were drawn with the *survival* package in R to assess the BCSS and OS rates, and log-rank tests were employed to examine statistical differences between subgroups. Univariable Cox proportional hazards regression was performed with the *survival* package to calculate the hazard ratios (HRs) and 95% CIs of each variable for BCSS and OS; multivariable Cox regression models were used to calculate the HRs and 95% CIs of BCSS and OS after adjusting for age, sex, race, tumor grade, T staging, N staging, histological type, HER2 status, surgery, chemotherapy, radiation therapy status, and HR status.

## Results

### Demographic and clinicopathological features

**Figure 1:**
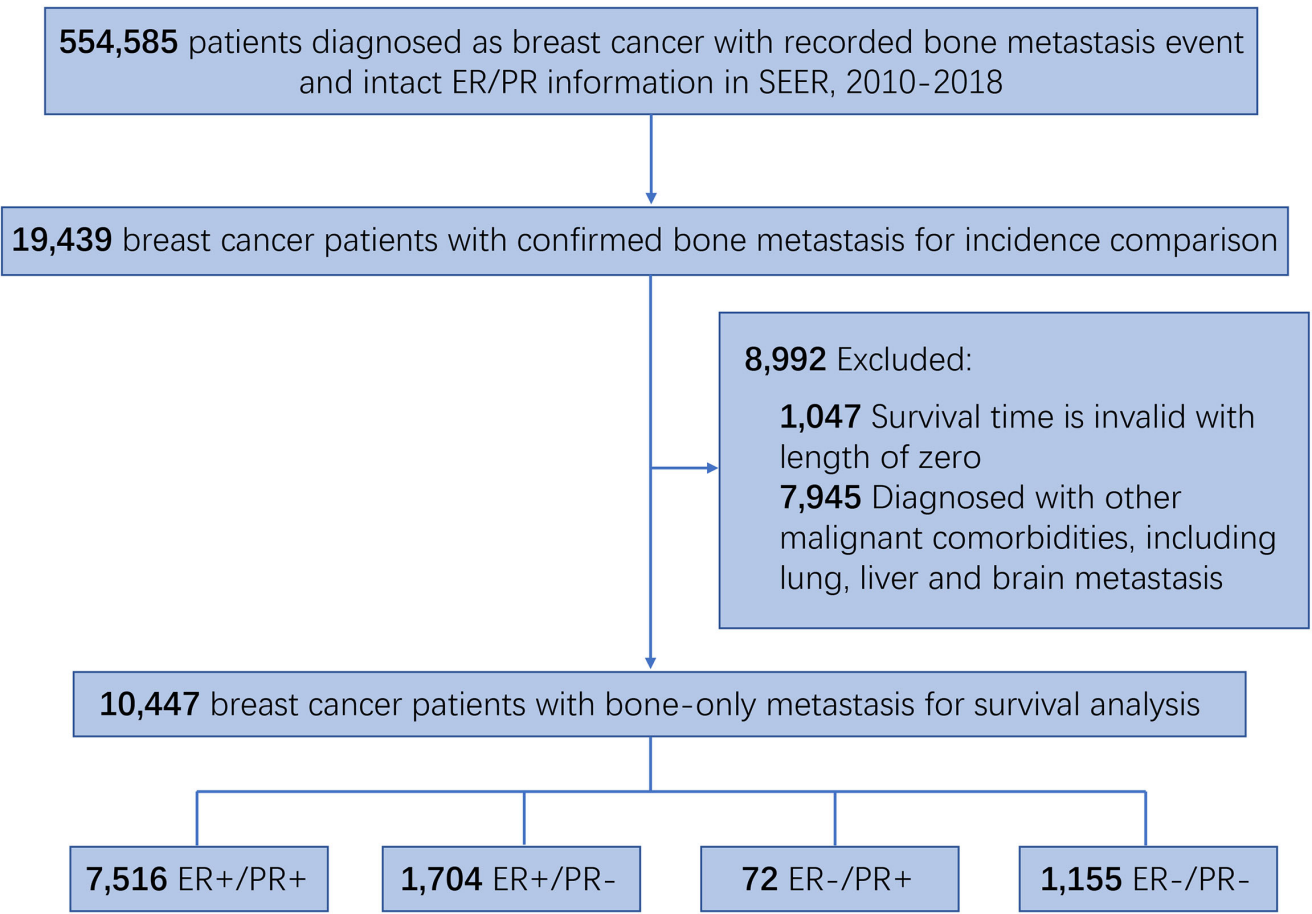
Flowchart of the cohort selection.

This retrospective cohort study consists of 554,585 breast cancer patients with recorded bone metastasis event and intact ER/PR information, among which 19,439 patients were confirmed with developing bone metastasis and 10,447 patients with bone-only metastasis and valid survival time (**Figure 1**). In order to investigate the differences in the incidence of bone metastasis across the four HR status breast cancers, the numbers and percentage of bone metastatic events were outlined (**Table 1**). Bone metastasis is most likely to develop in ER-positive/PR-negative breast cancers than in other subgroups, while the incidence of bone metastasis is similar between ER-positive/PR-positive and ER-negative/PR-negative breast cancers. Thereafter, to exclude the potential influence of other concurrent metastases, including brain, liver, or lung metastasis, we outlined the demographic and clinicopathological features of the 10,447 breast cancer patients with sole bone metastasis, which was included into the subsequent survival analysis (**Table 2**). Among the cohort, 71.9% (7,516/10,447), 16.3% (1,704/10,447), 0.7% (72/10,447), and 11.1% (1,155/10,447) of the bone metastatic breast cancer patients had ER-positive/PR-positive, ER-positive/PR-negative, ER-negative/PR-positive, and ER-negative/PR-negative primary tumors, respectively.

**Table 1:** Bone metastasis incidence in breast cancer patients stratified by hormone receptor status.

	All patients n (%) N = 554,585	ER+/PR+ n (%) N = 398,203	ER+/PR- n (%) N = 64,987	ER-/PR+ n (%) N = 5,548	ER-/PR- n (%) N = 85,847	P value
**Bone metastasis**						
Yes	19,439 (3.5%)	13,038 (3.3%)	3,216 (4.9%)	211 (3.8%)	2,974 (3.5%)	<0.001
No	535,146 (96.5%)	385,165 (96.7%)	61,771 (95.1%)	5,337 (96.2%)	82,873 (96.5%)	

From the SEER database, 2010–2018.

**Table 2:** Demographic and clinicopathological characteristics of breast cancer patients with bone metastasis stratified by hormone receptor status.

	All patients n (%) N = 10,447	ER+/PR+ n (%) N = 7,516	ER+/PR- n (%) N = 1,704	ER-/PR+ n (%) N = 72	ER-/PR- n (%) N = 1,155	P value
**Age**						
<49	2,053 (19.7%)	1,546 (20.6%)	239 (14.0%)	12 (16.7%)	256 (22.2%)	<0.001
50-69	5,194 (49.7%)	3,658 (48.7%)	906 (53.2%)	36 (50.0%)	594 (51.4%)	
>70	3,200 (30.6%)	2,312 (30.8%)	559 (32.8%)	24 (33.3%)	305 (26.4%)	
**Sex**						
Female	10,313 (98.7%)	7,405 (98.5%)	1,690 (99.2%)	72 (100%)	1146 (99.2%)	0.052
Male	134 (1.3%)	111 (1.5%)	14 (0.8%)	0 (0%)	9 (0.8%)	
**Race**						
White	8,181 (78.3%)	5,964 (79.4%)	1,338 (78.5%)	52 (72.2%)	827 (71.6%)	<0.001
Black	1,462 (14.0%)	958 (12.7%)	255 (15.0%)	12 (16.7%)	237 (20.5%)	
Other	766 (7.3%)	565 (7.5%)	107 (6.3%)	8 (11.1%)	86 (7.4%)	
Unknown	38 (0.4%)	29 (0.4%)	4 (0.2%)	0 (0%)	5 (0.4%)	
**Tumor grade**						
I	912 (8.7%)	784 (10.4%)	113 (6.6%)	0 (0%)	15 (1.3%)	<0.001
II	3,739 (35.8%)	2,948 (39.2%)	551 (32.3%)	16 (22.2%)	224 (19.4%)	
III	2,689 (25.7%)	1,636 (21.8%)	433 (25.4%)	30 (41.7%)	590 (51.1%)	
IV	18 (0.2%)	9 (0.1%)	5 (0.3%)	0 (0%)	4 (0.3%)	
Unknown	3,089 (29.6%)	2,139 (28.5%)	602 (35.3%)	26 (36.1%)	322 (27.9%)	
**T stage**						
T1	1,060 (10.1%)	773 (10.3%)	187 (11.0%)	5 (6.9%)	95 (8.2%)	<0.001
T2	2,108 (20.2%)	1,599 (21.3%)	307 (18.0%)	10 (13.9%)	192 (16.6%)	
T3	1,037 (9.9%)	747 (9.9%)	167 (9.8%)	7 (9.7%)	116 (10.0%)	
T4	6,242 (59.7%)	4,397 (58.5%)	1,043 (61.2%)	50 (69.4%)	752 (65.1%)	
**N stage**						
N0	1,849 (17.7%)	1,355 (18.0%)	316 (18.5%)	9 (12.5%)	169 (14.6%)	0.008
N1	2,711 (26.0%)	1,974 (26.3%)	409 (24.0%)	21 (29.2%)	307 (26.6%)	
N2	721 (6.9%)	520 (6.9%)	111 (6.5%)	7 (9.7%)	83 (7.2%)	
N3	856 (8.2%)	577 (7.7%)	148 (8.7%)	6 (8.3%)	125 (10.8%)	
Unknown	4,310 (41.3%)	3,090 (41.1%)	720 (42.3%)	29 (40.3%)	471 (40.8%)	
**Histological type**						
IDC	6,604 (63.2%)	4,758 (63.3%)	956 (56.1%)	54 (75.0%)	836 (72.4%)	<0.001
ILC	1,831 (17.5%)	1,389 (18.5%)	351 (20.6%)	2 (2.8%)	89 (7.7%)	
Mixed IDC and ILC	596 (5.7%)	469 (6.2%)	79 (4.6%)	1 (1.4%)	47 (4.1%)	
Other	1,416 (13.6%)	900 (12.0%)	318 (18.7%)	15 (20.8%)	183 (15.8%)	
**HER2 status**						
Positive	1,743 (16.7%)	918 (12.2%)	412 (24.2%)	29 (40.3%)	384 (33.2%)	<0.001
Negative	8,156 (78.1%)	6,177 (82.2%)	1,207 (70.8%)	36 (50.0%)	736 (63.7%)	
Borderline/unknown	548 (5.2%)	421 (5.6%)	85 (5.0%)	7 (9.7%)	35 (3.0%)	
**Surgery status**						
Yes	3,203 (30.7%)	2,250 (29.9%)	475 (27.9%)	27 (37.5%)	451 (39.0%)	<0.001
No	7,176 (68.7%)	5,218 (69.4%)	1,217 (71.4%)	45 (62.5%)	696 (60.3%)	
Unknown	68 (0.7%)	48 (0.6%)	12 (0.7%)	0 (0%)	8 (0.7%)	
**Chemotherapy**						
Yes	5,564 (53.3%)	3,668 (48.8%)	922 (54.1%)	51 (70.8%)	923 (79.9%)	<0.001
No or unknown	4,883 (46.7%)	3,848 (51.2%)	782 (45.9%)	21 (29.2%)	232 (20.1%)	
**Radiation therapy**						
Yes	3,921 (37.5%)	2,792 (37.1%)	653 (38.3%)	30 (41.7%)	446 (38.6%)	0.566
No or unknown	6,526 (62.5%)	4,724 (62.9%)	1,051 (61.7%)	42 (58.3%)	709 (61.4%)	
**Vital status**						
Alive	5,048 (48.3%)	3,882 (51.6%)	724 (42.5%)	19 (26.4%)	423 (36.6%)	<0.001
Dead of breast cancer	4,734 (45.3%)	3,159 (42.0%)	880 (51.6%)	45 (62.5%)	650 (56.3%)	
Dead of other cause	665 (6.4%)	475 (6.3%)	100 (5.9%)	8 (11.1%)	82 (7.1%)	

From the SEER database, 2010–2018.

Compared with other subgroups, bone metastatic breast cancer patients with ER-positive/PR-negative tumor tend to be older, whereas those with ER-positive/PR-positive and ER-negative/PR-negative ones were more frequently diagnosed in patients aged 49 years or younger; White race patients were more likely to have ER-positive tumor, while black race patients tended to have ER-negative tumors. ER-positive primary tumors were less aggressive, reflected by a lower tumor grade and T or N staging, while ER-negative primary tumors were more frequently diagnosed with higher tumor grade and advanced T or N staging, regardless of PR status. Moreover, ER-negative breast cancers were more commonly diagnosed with invasive ductal carcinoma (IDC), ER-negative ones with invasive lobular carcinoma (ILC). HER2-positive tumors were more common in PR-negative and ER-negative/PR-positive breast cancers. A higher proportion of ER-negative/PR-positive and ER-negative/PR-negative patients received surgery and chemotherapy than other subgroups, while acceptance of radiation therapy was equal across the four groups.

### Survival and prognosis analysis

**Figure 2:**
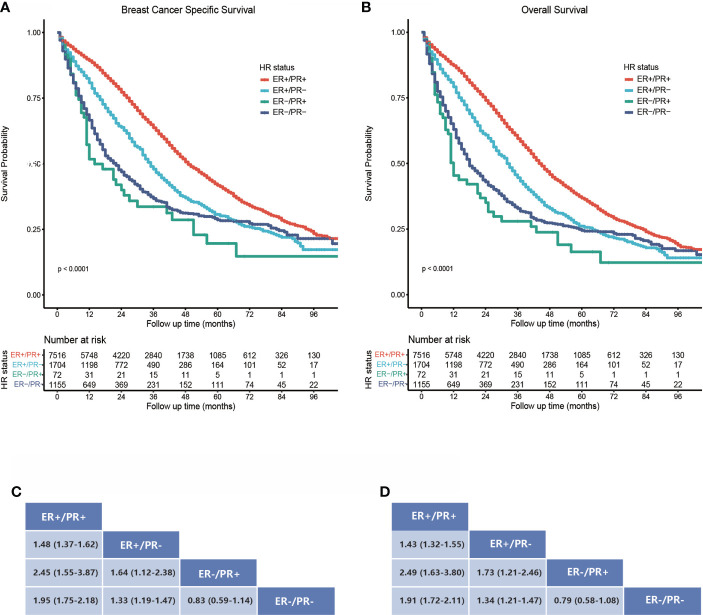
Survival analysis of bone metastatic breast cancer patients stratified by hormone receptor status. **(A)** Kaplan–Meier survival curves show a breast cancer-specific survival rate difference. **(B)** Kaplan–Meier survival curves show an overall survival rate difference. **(C, D)** Data are presented as hazard ratios (95% CIs) between HR status subgroups regarding BCSS and OS. A hazard ratio exceeding 1 favors the column-labeled HR status subgroup.

We compared the BCSS and OS difference within the four subgroups, which differed statistically significantly with unadjusted survival analysis (**Figures 2A, B**). The estimated median BCSS was 49 (95% CI: 48–51) months in ER-positive/PR-positive breast cancers, 35 (95% CI: 33–37) months in ER-positive/PR-negative breast cancers, 14 (95% CI: 11–30) months in ER-negative/PR-negative breast cancers, and 22 (95% CI: 18–25) months in ER-negative/PR-negative breast cancers, and the estimated median OS was 44 (95% CI: 43–46) months in ER-positive/PR-positive breast cancers, 32 (95% CI: 31–34) months in ER-positive/PR-negative breast cancers, 12 (95% CI: 11–24) months in ER-negative/PR-positive breast cancers, and 18 (95% CI: 17–21) months in ER-negative/PR-negative breast cancers. Compared with bone metastatic breast cancer patients who had ER-positive/PR-positive primary tumor, those with ER-positive/PR-negative (HR = 1.48, 95% CI: 1.37–1.62), ER-negative/PR-positive (HR = 2.45, 95% CI: 1.55–3.87), and ER-negative/PR-negative (HR = 1.95, 95% CI: 1.75–2.18) breast cancers showed poorer BCSS (all log-rank p < 0.001, **Figure 2C**). Moreover, patients with ER-positive/PR-negative bone metastatic breast cancer had significantly better BCSS than ER-negative/PR-positive (HR = 1.64, 95% CI: 1.12–2.38) and ER-negative/PR-negative (HR = 1.33, 95% CI: 1.19–1.47) breast cancers (both log-rank p < 0.001, **Figure 2C**). In contrast, there was no statistically significant difference in BCSS between ER-negative/PR-positive and ER-negative/PR-negative breast cancers (HR = 0.83, 95% CI: 0.59–1.14, log-rank p = 0.207, **Figure 2C**), and the same difference trend was found in OS of bone metastatic breast cancer subgroups with four HR statuses (**Figure 2D**).

We next performed univariate and multivariate Cox regression analyses to find out the statistical significant prognostic factors for breast cancer patients with bone metastases and further compared the survival between the four HR status subgroups with univariate analysis (**Table 3**). In the univariate analysis, patients with older age, male, black race, ILC, higher tumor grade, T3–T4, HER2-negative status, absence of surgery or adjuvant treatment, and HR status other than ER-positive/PR-positive were found to correlate with worse BCSS and OS. These statistically significant factors were then included in the multivariate analysis; patients aged 50 years or older, black race, higher tumor grade and T stage, HER2 negativity, absence of chemotherapy, and HR status other than ER-positive/PR-positive still related with worse BCSS and OS. Moreover, after adjusting for the covariates, compared with the ER-positive/PR-positive bone metastatic breast cancers, the ER-positive/PR-negative, ER-negative/PR-positive, and ER-negative/PR-negative subgroups were statistically associated with 51% (HR = 1.51, 95% CI: 1.40–1.64), 232% (HR = 3.32, 95% CI: 2.46–4.47), and 151% (HR = 2.51, 95% CI: 2.28–2.75) increased risks of breast cancer specific death respectively, and 47% (HR = 1.47, 95% CI: 1.37–1.58), 241% (HR = 3.41, 95% CI: 2.59–4.49), and 148% (HR = 2.48, 95% CI: 2.27–2.71) increased risks of overall death.

**Table 3:** Univariate and multivariate Cox proportional hazard regression of OS and BCSS in breast cancer patients with bone metastasis.

	OS				BCSS			
	Univariate analysis		Multivariate analysis		Univariate analysis		Multivariate analysis	
	HR (95% CI)	P value	HR (95% CI)	P value	HR (95% CI)	P value	HR (95% CI)	P value
**Age**								
<50	Reference				Reference			
50-69	1.44 (1.33-1.55)	<0.001	1.30 (1.20-1.40)	<0.001	1.39 (1.28-1.51)	<0.001	1.25 (1.15-1.36)	<0.001
>70	2.37 (2.19-2.57)	<0.001	1.95 (1.79-2.12)	<0.001	2.12 (1.95-2.31)	<0.001	1.76 (1.60-1.92)	<0.001
**Sex**								
Female	Reference				Reference			
Male	1.32 (1.05-1.65)	0.017	1.39 (1.11-1.75)	0.004	1.30 (1.02-1.66)	0.033	1.41 (1.11-1.81)	0.005
**Race**								
White	Reference				Reference			
Black	1.37 (1.28-1.48)	<0.001	1.35 (1.25-1.45)	<0.001	1.39 (1.29-1.51)	<0.001	1.35 (1.25-1.46)	<0.001
Other	0.89 (0.78-0.99)	0.041	0.96 (0.86-1.08)	0.55	0.92 (0.82-1.04)	0.187	0.99 (0.88-1.11)	0.903
Unknown	1.09 (0.60-1.97)	0.779	0.98 (0.54-1.77)	0.953	1.03 (0.53-1.98)	0.936	0.90 (0.46-1.74)	0.763
**Histological type**								
IDC	Reference				Reference			
ILC	1.21 (1.13-1.30)	<0.001	1.15 (1.07-1.24)	<0.001	1.25 (1.16-1.34)	<0.001	1.21 (1.12-1.31)	<0.001
Mixed IDC and ILC	0.98 (0.87-1.11)	0.789	1.06 (0.94-1.19)	0.306	1.01 (0.89-1.15)	0.849	1.11 (0.97-1.25)	0.103
Other	1.33 (1.23-1.44)	<0.001	1.05 (0.97-1.14)	0.201	1.34 (1.23-1.45)	<0.001	1.06 (0.97-1.16)	0.161
**Tumor grade**								
I	Reference				Reference			
II	1.15 (1.03-1.27)	0.01	1.20 (1.08-1.34)	<0.001	1.18 (1.06-1.32)	0.003	1.25 (1.11-1.40)	<0.001
III	1.55 (1.39-1.72)	<0.001	1.72 (1.54-1.92)	<0.001	1.63 (1.46-1.83)	<0.001	1.81 (1.61-2.05)	<0.001
IV	2.69 (1.58-4.58)	<0.001	2.85 (1.67-4.87)	<0.001	3.20 (1.88-5.46)	<0.001	3.22 (1.88-5.52)	<0.001
Unknown	1.60 (1.44-1.79)	<0.001	1.31 (1.17-1.46)	<0.001	1.67 (1.48-1.87)	<0.001	1.37 (1.21-1.54)	<0.001
**T stage**								
T1	Reference				Reference			
T2	1.05 (0.96-1.15)	0.302	1.17 (1.06-1.29)	0.001	1.05 (0.95-1.16)	0.324	1.16 (1.05-1.29)	0.003
T3	1.17 (1.05-1.30)	0.004	1.22 (1.09-1.36)	<0.001	1.19 (1.06-1.34)	0.003	1.22 (1.09-1.38)	<0.001
T4	1.39 (1.27-1.52)	<0.001	1.32 (1.20-1.44)	<0.001	1.40 (1.28-1.54)	<0.001	1.32 (1.20-1.46)	<0.001
**N stage**								
N0	Reference				Reference			
N1	0.94 (0.88-1.01)	0.116	1.02 (0.94-1.10)	0.572	0.96 (0.89-1.03)	0.26	1.02 (0.94-1.10)	0.538
N2	0,94 (0.84-1.04)	0.241	1.18 (1.06-1.32)	0.002	0.97 (0.87-1.09)	0.611	1.21 (1.07-1.36)	0.001
N3	1.07 (0.97-1.18)	0.177	1.30 (1.17-1.44)	<0.001	1.11 (1.00-1.22)	0.053	1.32 (1.19-1.48)	<0.001
Unknown	1.11 (1.02-1.21)	0.01	1.03 (0.95-1.13)	0.401	1.10 (1.01-1.20)	0.028	1.01 (0.92-1.11)	0.763
**HER2 status**								
Positive	Reference				Reference			
Negative	1.49 (1.37-1.61)	<0.001	1.67 (1.53-1.82)	<0.001	1.49 (1.37-1.62)	<0.001	1.72 (1.57-1.89)	<0.001
Borderline/unknown	1.70 (1.50-1.93)	<0.001	1.62 (1.41-1.85)	<0.001	1.69 (1.47-1.93)	<0.001	1.66 (1.44-1.91)	<0.001
**Surgery status**								
Yes	Reference				Reference			
No	1.75 (1.65-1.86)	<0.001	1.78 (1.66-1.91)	<0.001	1.74 (1.63-1.86)	<0.001	1.83 (1.70-1.96)	<0.001
Unknown	1.76 (1.27-2.43)	<0.001	1.82 (1.32-2.52)	<0.001	1.89 (1.36-2.64)	<0.001	2.03 (1.46-2.84)	<0.001
**Radiation therapy**								
Yes	Reference				Reference			
No or unknown	1.17 (1.11-1.23)	<0.001	0.99 (0.93-1.05)	0.808	1.12 (1.06-1.19)	<0.001	0.95 (0.89-1.01)	0.126
**Chemotherapy**								
Yes	Reference				Reference			
No or unknown	1.48 (1.41-1.57)	<0.001	1.36 (1.28-1.45)	<0.001	1.42 (1.34-1.50)	<0.001	1.32 (1.24-1.41)	<0.001
**HR status**								
ER+/PR+	Reference				Reference			
ER+/PR-	1.43 (1.33-1.53)	<0.001	1.47 (1.37-1.58)	<0.001	1.48 (1.37-1.59)	<0.001	1.51 (1.40-1.64)	<0.001
ER-/PR+	2.47 (1.88-3.24)	<0.001	3.41 (2.59-4.49)	<0.001	2.42 (1.81-3.25)	<0.001	3.32 (2.46-4.47)	<0.001
ER-/PR-	1.92 (1.78-2.08)	<0.001	2.48 (2.27-2.71)	<0.001	1.97 (1.81-2.14)	<0.001	2.51 (2.28-2.75)	<0.001

From the SEER database, 2010–2018.

### Subgroup analysis based on HER2 status and other features

ERBB2 gene amplification or HER2 overexpression is prevalent in approximately 20% of breast cancers, and heterogeneity also exists within this subtype. The varied HR status in HER2-positive breast cancers contributes to their distinct biological behavior and treatment response. Ample evidence has pointed out that ER-positive/HER2-positive breast cancers are more likely to metastasize to bone, while studies regarding survival outcome between various HR statuses in HER2-positve or negative breast cancer with bone metastasis are limited till now. Here we further performed subgroup analysis based on HER2 status, as well as other prognosis-related features, to dissect the survival difference.

**Figure 3:**
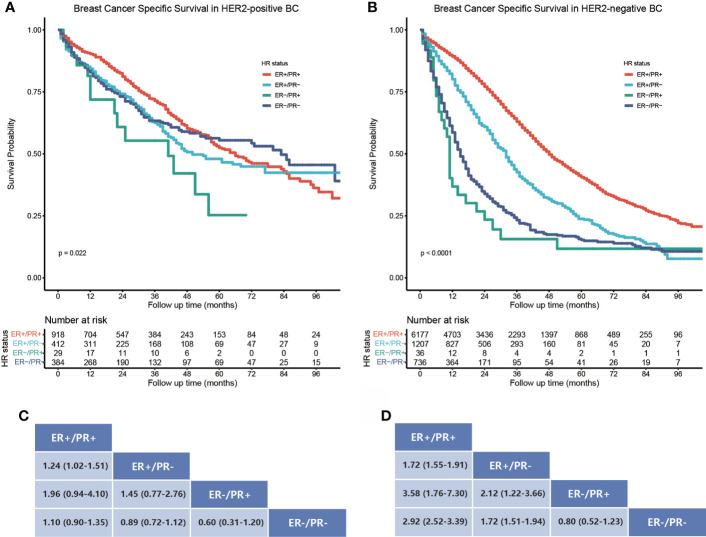
Subgroup BCSS analysis in HER2-positive and negative bone metastatic breast cancers stratified by HR status. **(A)** Kaplan–Meier survival curves show the BCSS rate difference in HER2-positive bone metastatic breast cancers stratified by HR status. **(B)** Kaplan–Meier survival curves show the BCSS rate difference in HER2-negative bone metastatic breast cancers stratified by HR status. **(C, D)** Data are presented as hazard ratios (95% CIs) between HR status subgroups regarding BCSS in HER2-positive and negative bone metastatic breast cancers. A hazard ratio exceeding 1 favors the column-labeled HR status subgroup.

**Figure 4:**
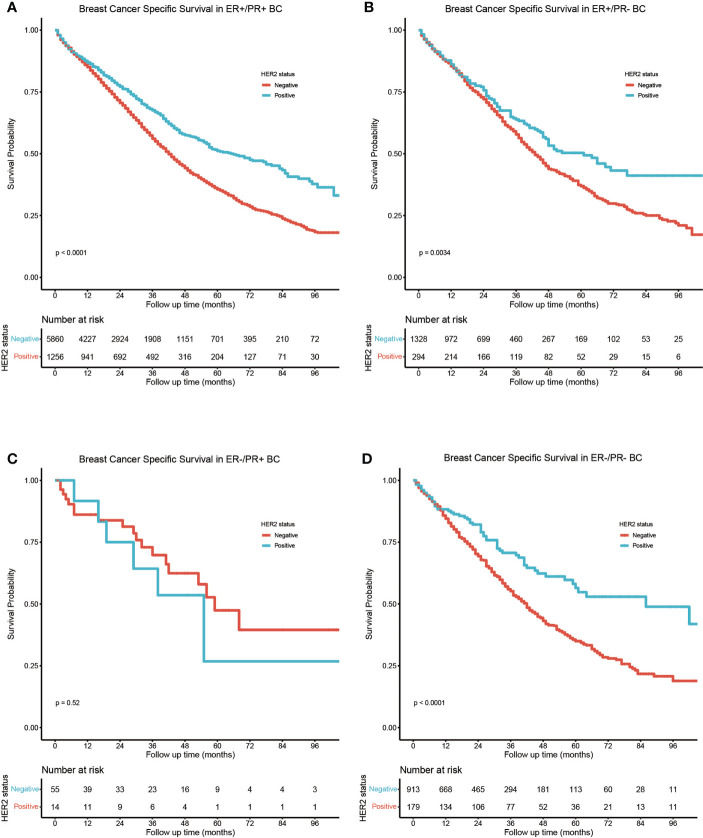
Subgroup BCSS analysis in **(A)** ER-positive/PR-positive, **(B)** ER-positive/PR-negative, **(C)** ER-negative/PR-positive, and **(D)** ER-negative/PR-negative bone metastatic breast cancers stratified by HER2 status.

The study cohort for survival analysis comprised 1,743 (16.7%) HER2-positive and 8,156 (78.1%) HER2-negative bone metastatic breast cancers; the HER2 status of borderline/unknown was excluded from the subgroup analysis. In HER2-positive bone metastatic breast cancers, the BCSS was best in the ER-positive/PR-positive subtype (**Figure 3A**), ER-positive/PR-negative (HR = 1.24, 95% CI: 1.02–1.51), and ER-negative/PR-positive (HR = 1.96, 95% CI: 0.94–4.10) breast cancers which manifested poorer BCSS than the ER-positive/PR-positive ones, while the BCSS difference between ER-positive/PR-negative, ER-negative/PR-positive, and ER-negative/PR-negative breast cancers was not statistically significant (**Figure 3C**). In HER2-negative bone metastatic breast cancers, the BCSS was still best in the ER-positive/PR-positive breast cancers (**Figure 3B**); the ER-positive/PR-negative (HR = 1.72, 95% CI: 1.55–1.91), ER-negative/PR-positive (HR = 3.58, 95% CI: 1.76–7.30), and ER-negative/PR-negative (HR = 2.92, 95% CI: 2.52–3.39) breast cancers manifested poorer BCSS (**Figure 3D**). BCSS in the ER-positive/PR-negative subgroup was also better than ER-negative/PR-positive (HR = 2.12, 95% CI: 1.22–3.66) and ER-negative/PR-negative ones (HR = 1.72, 95% CI: 1.51–1.94). Also, the BCSS difference was statistically significant between HER2-positive and HER2-negative subgroups in ER-positive/PR-positive, ER-positive/PR-negative, and ER-negative/PR-negative breast cancers (**Figures 4A, B, D**), except for the ER-negative/PR-positive subtype (**Figure 4C**). Taken together, the ER-positive/PR-positive bone metastatic breast cancers manifest the best BCSS.

The same trend in which BCSS was best in the ER-positive/PR-positive subgroup than in the ER-positive/PR-negative, ER-negative/PR-positive, and ER-negative/PR-negative subgroups was still observed in subgroup analysis stratified by age, sex, race, tumor grade, T stage, histological type, acceptance of surgery, chemotherapy, or radiotherapy (**Figures S1**–**S9** in the (**Supplementary Figures**).

## Discussion

Owing to the huge heterogeneity in breast cancer intrinsic characteristics and metastatic pattern, proper stratification should be performed, which can guide tailored treatment strategies (18, 19). It is well recognized that HR-positive breast cancers are prone to develop bone metastasis, and accumulating preclinical evidence has demonstrated that steroid hormones and their HR signaling participate tightly in the process of bone metastasis formation (20, 21). To uncover the contribution of HR status to bone metastasis and its potential impact on survival outcome in real-world cohorts, the current study investigated the four kinds of HR status of breast cancer regarding their bone metastasis prevalence, clinicopathological features, and survival outcome comparison. Notably, a differed HR status was validated as the prognostic significant factor in breast cancer bone metastasis, and the results may be significant for risk stratification in breast cancer bone metastasis and guide tailored treatment.

Breast cancer bone metastases are heterogenous lytic and sclerotic lesions, predominated by osteolytic foci with minor osteoblastic areas (4). The imbalanced bone metabolism caused by strengthened osteoclast activity leads to formation of a low-density bone microenvironment, which is the indispensable pre-metastatic niche which facilitates cancer cell colonization (22–24). Briefly, after seeding in bone, cancer cells would secrete parathyroid hormone-related protein (PTHrP) to stimulate receptor activator for nuclear factor-κB ligand (RANKL) expression on osteoblasts and further bind with RANK on osteoclasts to signify bone resorption. Various growth factors embedded in the bone matrix are also released accompanying this process and benefit seeded cancer cell survival and propagation subsequently, which forms a vicious cycle to create fertile soil to promote aggressive behavior (25). In recent years, accumulating evidence has revealed that hormone and HR under tumoral conditions facilitate cancer bone metastasis *via* participating in this process. Chiu et al. demonstrated that estrogens upregulate E-cadherin in metastatic breast cancer cells to endow them with epithelial-like properties and facilitate seeding. Also, the ER-Src-p190RhoGAP axis is responsible for enhanced cancer cell proliferation and attenuated migration, which also favors colonization of bone tissue (21). In MCF-7 cell-inoculated mice, Cheng et al. found that bone metastases could not form in the absence of estrogen; while supplementation with estrogen led to increased incidence and size of bone metastases in a dose-dependent manner, the osteoclast number and secretion of PTHrP are also upregulated by estrogen administration mediated by ERα, indicating that ER may serve as not only a biomarker but also a potential molecular driver in osteolysis and metastatic progression in breast cancer (26). Ogba et al. also discovered that ER-positive/PR-positive breast cancer cell-inoculated ovary-excised mice remained metastasis-free until they were supplemented with estrogen or estrogen plus progestin (27), suggesting that hormone signaling promotes bone metastases in luminal breast cancer. All the above findings jointly demonstrate that steroid hormones could promote bone metastasis progression potentially *via* HR signaling.

Despite that HR signaling has been proved to participate in bone metastasis, varied ER and PR statuses in breast cancer have been demonstrated to associate with distinct characteristics and survival and thus may exert different bone metastasis-forming capacities and lead to different survival outcomes. Although previously collectively regarded as HR-positive breast cancer, single HR-positive breast cancer has been proven to be a distinct subgroup from double HR-positive or negative breast cancer (28, 29). Single HR-positive breast cancers, i.e., ER-positive/PR-negative or ER-negative/PR-positive ones, have worse survival than ER-positive/PR-positive breast cancer, but better survival than ER-negative/PR-negative breast cancer, while survival in single ER-positive breast cancer is longer than that in single PR-positive breast cancer (29). In addition, ER-positive/PR-negative breast cancer patients tend to be older and manifest a more aggressive behavior, with larger tumor size, lymph node positivity, higher histological grade, and shortened breast cancer-specific survival (BCSS). In contrast, ER-negative/PR-positive breast cancers are diagnosed in the younger population and are less aggressive. Since PR expression is dependent on ER signaling pathways, ER-positive/PR-positive is the most common HR status in breast cancer and confers the most sensitive response to endocrine therapy (30). In contrast, PR loss, leading to a single ER-positive subpopulation, is prevalent in 6.9%–15.6% breast cancers, which could be attributed to multiple causes (31). PR loss in breast cancer confers resistance to hormone treatment but better response to chemotherapy (31). A reduced estrogen level in the elderly leads to a lower expression of PGR, which is one ERα-dependent gene and encodes PR, which could explain the higher prevalence of PR loss in older patients. ER loss in breast cancer is a rare event, and the presence of single PR-positive breast cancer is under debate since PGR is the downstream responsive gene. Insensitive detection of low ER expression leading to immunohistochemical staining discrepancy and ESR mutation may cause decreased ligand-binding affinity, which could explain the emergence of single PR-positive breast cancer (32), while this subgroup still exists even after excluding these potential causes, and consistency of the recorded incidence confirm the presence of single PR-positive breast cancer. Multiple growth factor signaling, including epithelial growth factor (EGF), and insulin-like growth factor (IGF) could interfere with PR signaling and regulate its expression (33), indicating that ER-negative/PR-positive is a distinct entity and proved to be morphologically and molecularly close to triple-negative breast cancer.

In this study, we compared both BCSS and OS between ER-positive/PR-positive, ER-positive/PR-negative, ER-negative/PR-positive, and ER-negative/PR-negative breast cancers with bone metastases, indicating that metastasis to the same organ confers different rates of survival depending on the HR status of breast cancer. In addition, we identified several features which associated with worse survival in bone metastatic breast cancer, including older age at diagnosis (>70 years old), higher tumor grade and T staging (T3–T4), HER2 negativity, absence of surgery and chemotherapy, and more importantly, primary tumor HR status of ER-negative/PR-positive or ER-negative/PR-negative. Bone metastatic breast cancer patients with ER-positive/PR-positive tumors demonstrated the best outcome among the four subgroups, and single ER-positive breast cancers presented preferred survival than those with single PR-positive tumors. However, no significant survival difference was detected between ER-negative/PR-positive and ER-negative/PR-negative bone metastatic breast cancer, which suggested that aside from their morphological and molecular similarity (34), they also resemble in aspect of the survival outcome in breast cancer with bone metastases.

HER2-positive breast cancers represent a significant breast cancer subtype, while recent research has revealed that certain heterogeneities also exist within this entity, and distinct HR statuses may contribute mainly to different treatment responses, metastatic patterns, and survival outcomes (35, 36). ER-positive/HER2-positive breast cancers are more prone to form bone metastases, instead of ER-negative/HER-2 positive ones which manifest a higher propensity to visceral organs including the brain, lung, and liver (37). In addition, either in primary or metastatic breast cancers, ER-negative/HER2-positive breast cancers have shown poor survival than ER-positive/HER2-positive ones (37), suggesting that the outcome of bone metastatic breast cancer may be dependent on both HR and HER2 statuses of the primary tumor. In our analysis, we found that HER2 status was a significant prognostic feature; therefore, we further performed subgroup analysis based on HER2 status, to explore the survival difference in HER2-positive and HER2-negative bone metastatic breast cancers with various HR statuses. Within the HER2-positive subtype, ER-positive/PR-positive breast cancers presented the best BCSS than other groups, while the survival outcome between the other three groups was not statistically significant. In the HER2-negative subtype, the BCSS difference was more evident, with ER-positive/PR-positive breast cancers still manifesting the most preferred survival among the four groups and ER-positive/PR-negative breast cancers showing a better outcome than ER-negative/PR-positive ones.

Several limitations need to be addressed in our study. Firstly, bone metastatic breast cancer patients with other malignant comorbidities or with invalid survival time were excluded to eliminate the potential effect to survival analysis, while potential selection bias could not be avoided. In addition, several important features, including confounding prognostic factors like complications, detailed treatments, and treatment duration, are unavailable in the SEER database, which might dampen the applicability of our findings in the real-world cases.

## Conclusion

In conclusion, our study demonstrated that different ER and PR statuses in breast cancer exert a significant impact on bone metastasis incidence and survival condition. Moreover, the HR status in bone metastatic breast cancer is a significant prognostic factor which determines the survival probability. The results of our analysis suggest that clinicians may need to take care on discriminating bone metastatic breast cancers based on their HR status, and HER2 status occasionally, before deciding on the intensity of treatment on breast cancer patients with bone metastases.

## Data availability statement

The original contributions presented in the study are included in the article/**Supplementary Material**. Further inquiries can be directed to the corresponding author.

## Author contributions

Conceptualization, XJ, GC, and CaL; formal analysis, YZ; funding acquisition, CaL; methodology, XJ, and LS; software, ChL; validation, ChL and ML; visualization, XJ; writing—original draft, XJ; writing—review and editing, GC, LS, and CaL. All authors have read and agreed to the published version of the manuscript.

## Funding

This research was funded by the National Natural Science Foundation of China, grant number 81572609.

## Acknowledgments

The authors acknowledge the efforts of the Surveillance, Epidemiology, and End Results (SEER) program registries in the creation of the SEER database.

## Conflict of interest

The authors declare that the research was conducted in the absence of any commercial or financial relationships that could be construed as a potential conflict of interest.

## Publisher’s note

All claims expressed in this article are solely those of the authors and do not necessarily represent those of their affiliated organizations, or those of the publisher, the editors and the reviewers. Any product that may be evaluated in this article, or claim that may be made by its manufacturer, is not guaranteed or endorsed by the publisher.

## Supplementary material

Supplementary Figure 1 Breast cancer-specific survival of bone metastatic breast cancer patients stratified by age, **(A)** younger than 49, **(B)** between 50 and 70, and **(C)** older than 70.

Supplementary Figure 2 Breast cancer-specific survival of bone metastatic breast cancer patients stratified by sex, **(A)** Female, **(B)** Male.

Supplementary Figure 3 Breast cancer-specific survival of bone metastatic breast cancer patients stratified by race, **(A)** Black race, **(B)** White race, and **(C)** Other race.

Supplementary Figure 4 Breast cancer-specific survival of bone metastatic breast cancer patients stratified by tumor grade, **(A)** Grade 1, **(B)** Grade 2, **(C)** Grade 3, and **(D)** Grade 4.

Supplementary Figure 5 Breast cancer-specific survival of bone metastatic breast cancer patients stratified by T stage, **(A)** T1, **(B)** T2, **(C)** T3, and **(D)** T4.

Supplementary Figure 6 Breast cancer-specific survival of bone metastatic breast cancer patients stratified by histological type, **(A)** IDC, **(B)** ILC, and **(C)** Mixed IDC and ILC.

Supplementary Figure 7 Breast cancer-specific survival of bone metastatic breast cancer patients stratified by acceptance of surgery, **(A)** accept surgery, **(B)** no surgery.

Supplementary Figure 8 Breast cancer-specific survival of bone metastatic breast cancer patients stratified by acceptance of chemotherapy, **(A)** accept chemotherapy, **(B)** no chemotherapy.

Supplementary Figure 9 Breast cancer-specific survival of bone metastatic breast cancer patients stratified by acceptance of radiotherapy, **(A)** accept radiotherapy, **(B)** no radiotherapy.
