# Supplementary figures and images for: Characteristics and survival in bone metastatic breast cancer patients with different hormone receptor status: A population-based cohort study
Source: Front Oncol. 2022 Aug 26;12:977226. doi: 10.3389/fonc.2022.977226 (PMC9459168; doi:10.3389/fonc.2022.977226)

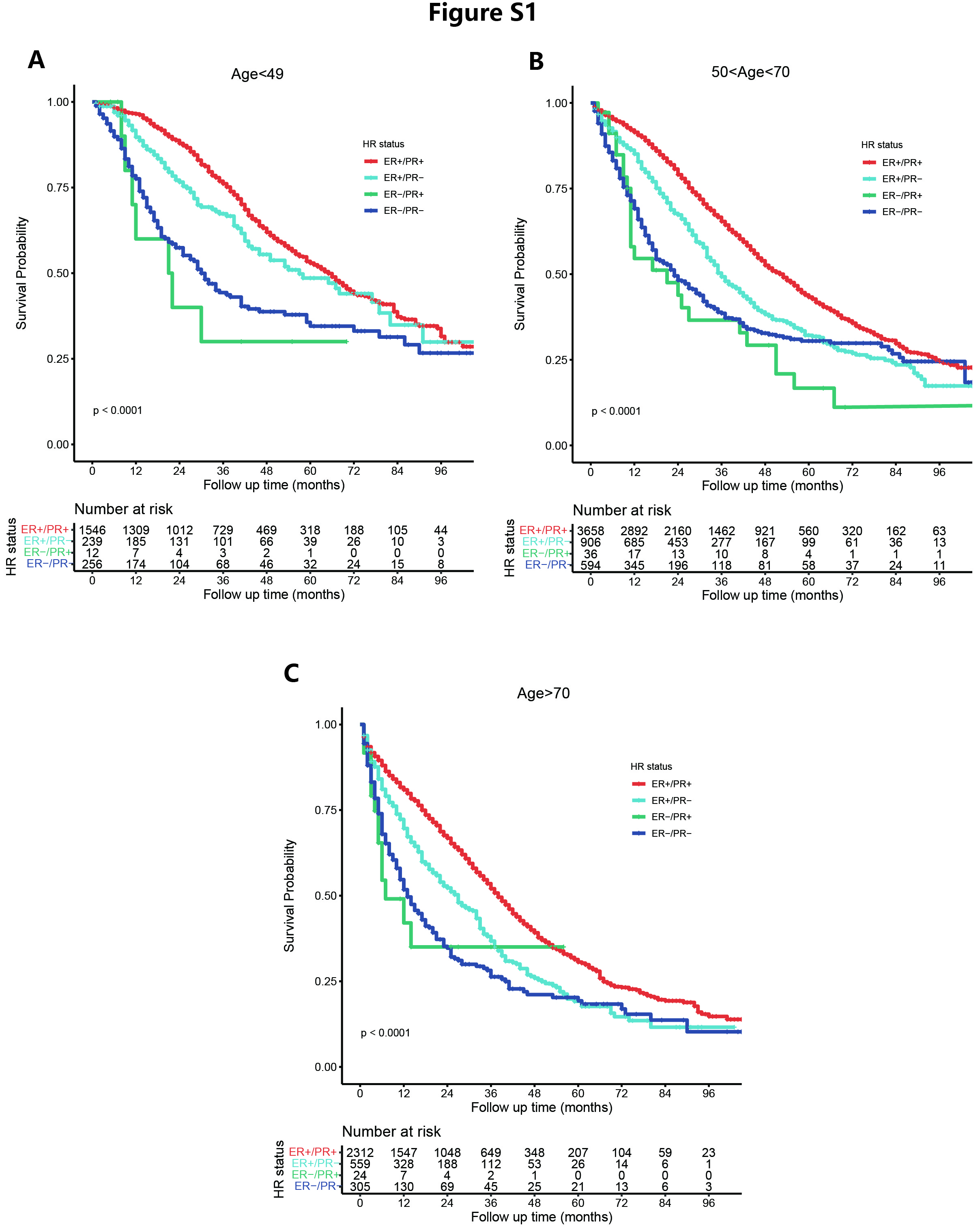

Supplement: Supplementary Figure 1 — Breast cancer-specific survival of bone metastatic breast cancer patients stratified by age, (A) younger than 49, (B) between 50 and 70, and (C) older than 70. [file Image_1.jpeg]

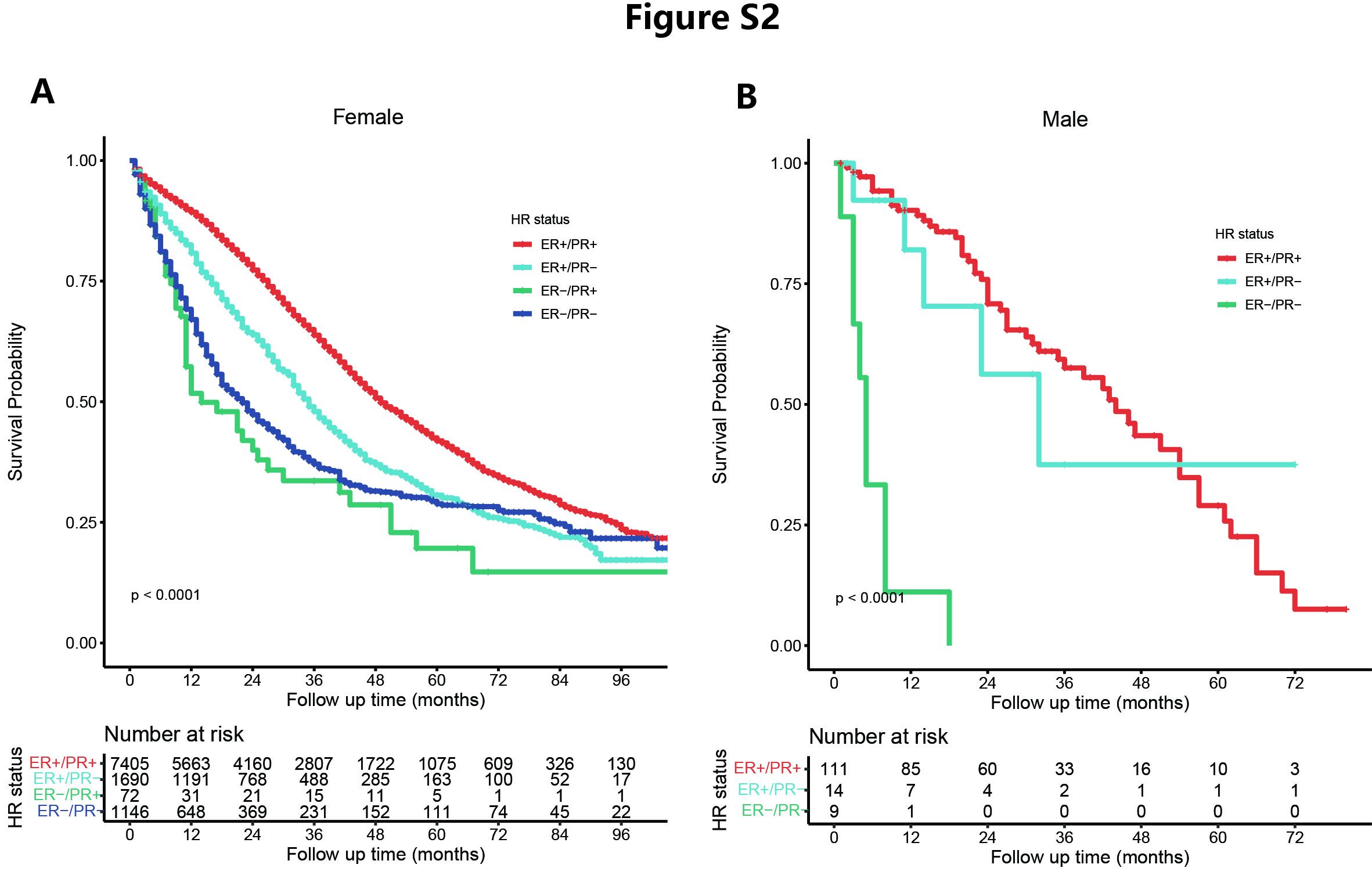

Supplement: Supplementary Figure 2 — Breast cancer-specific survival of bone metastatic breast cancer patients stratified by sex, (A) Female, (B) Male. [file Image_2.jpeg]

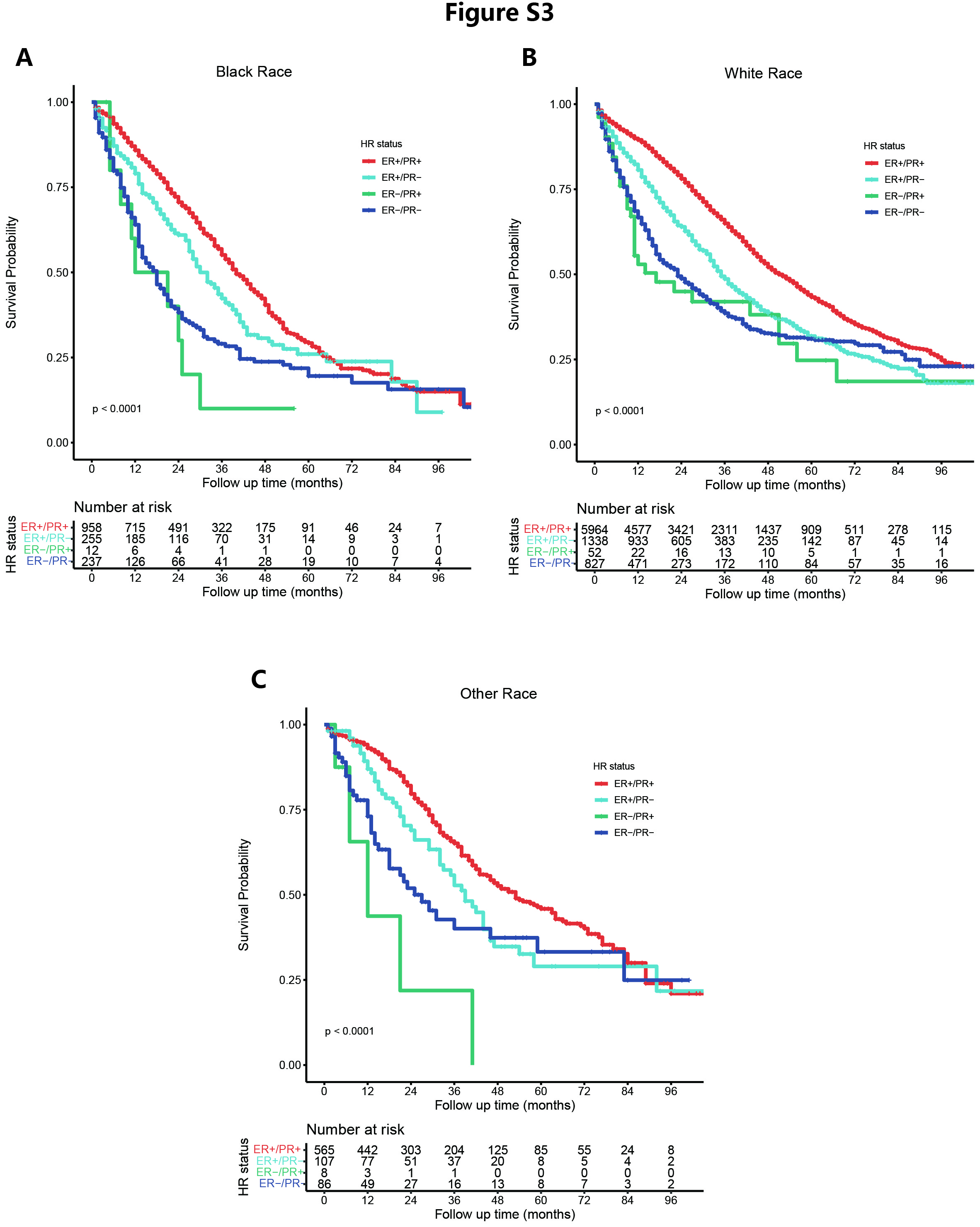

Supplement: Supplementary Figure 3 — Breast cancer-specific survival of bone metastatic breast cancer patients stratified by race, (A) Black race, (B) White race, and (C) Other race. [file Image_3.jpeg]

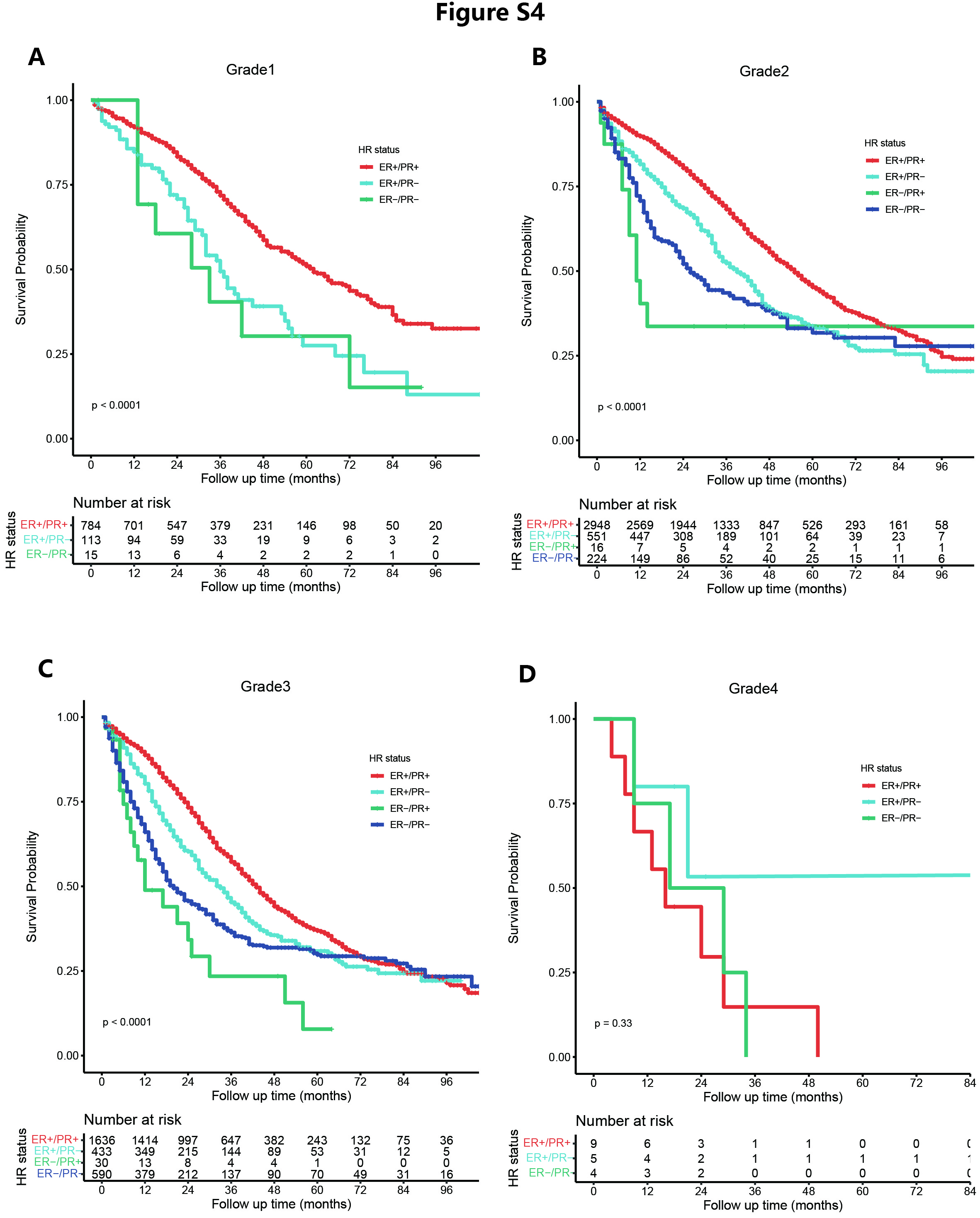

Supplement: Supplementary Figure 4 — Breast cancer-specific survival of bone metastatic breast cancer patients stratified by tumor grade, (A) Grade 1, (B) Grade 2, (C) Grade 3, and (D) Grade 4. [file Image_4.jpeg]

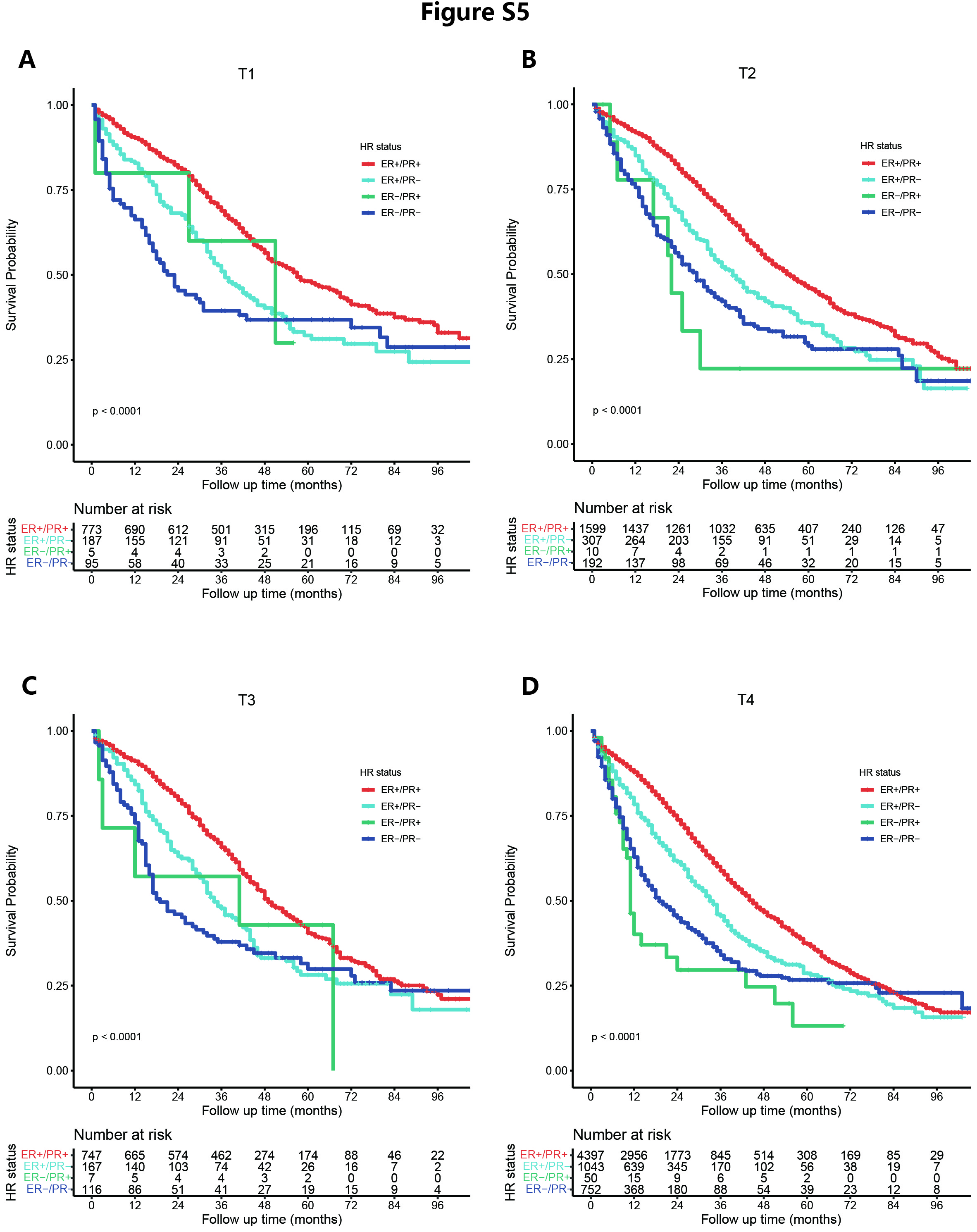

Supplement: Supplementary Figure 5 — Breast cancer-specific survival of bone metastatic breast cancer patients stratified by T stage, (A) T1, (B) T2, (C) T3, and (D) T4. [file Image_5.jpeg]

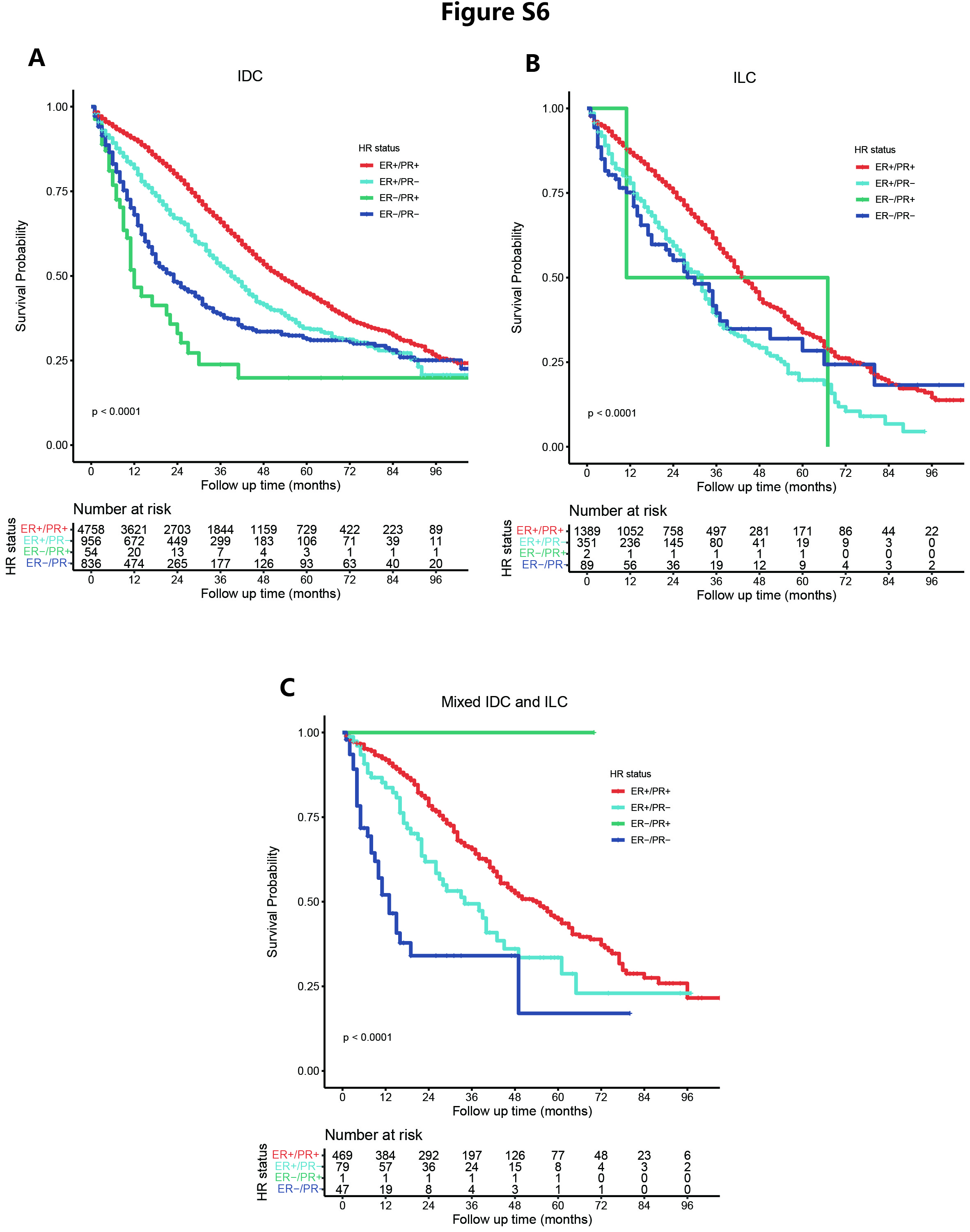

Supplement: Supplementary Figure 6 — Breast cancer-specific survival of bone metastatic breast cancer patients stratified by histological type, (A) IDC, (B) ILC, and (C) Mixed IDC and ILC. [file Image_6.jpeg]

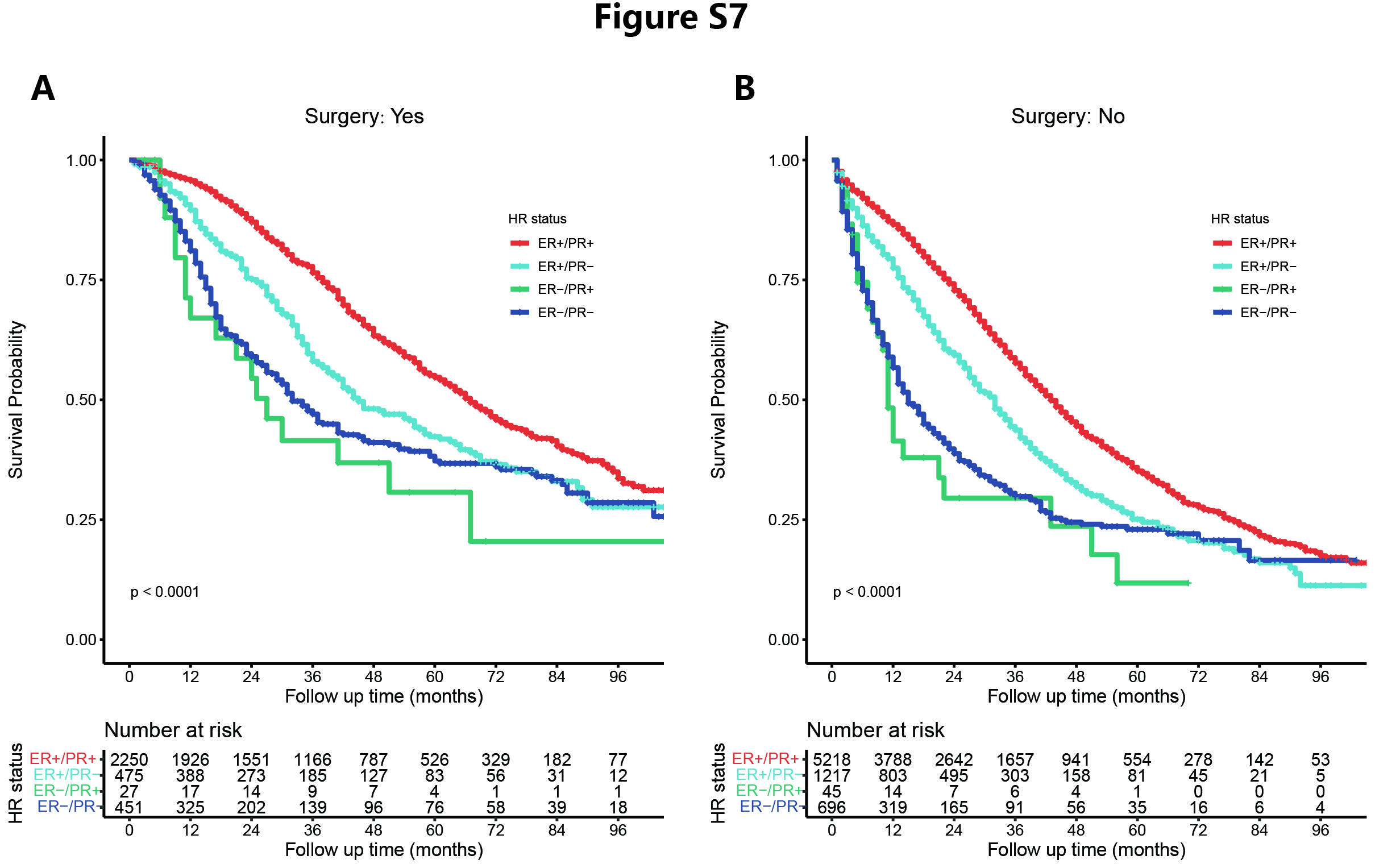

Supplement: Supplementary Figure 7 — Breast cancer-specific survival of bone metastatic breast cancer patients stratified by acceptance of surgery, (A) accept surgery, (B) no surgery. [file Image_7.jpeg]

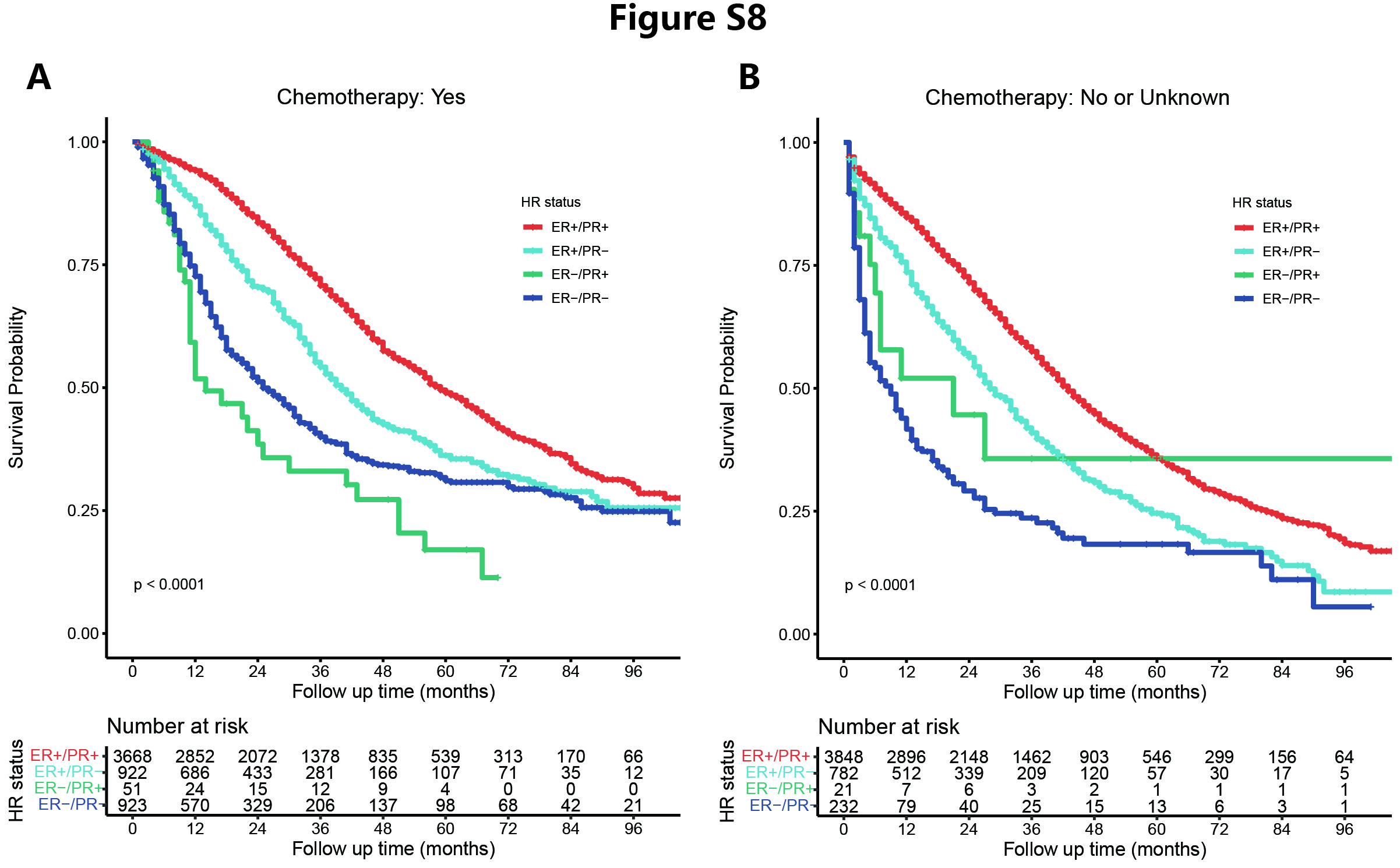

Supplement: Supplementary Figure 8 — Breast cancer-specific survival of bone metastatic breast cancer patients stratified by acceptance of chemotherapy, (A) accept chemotherapy, (B) no chemotherapy. [file Image_8.jpeg]

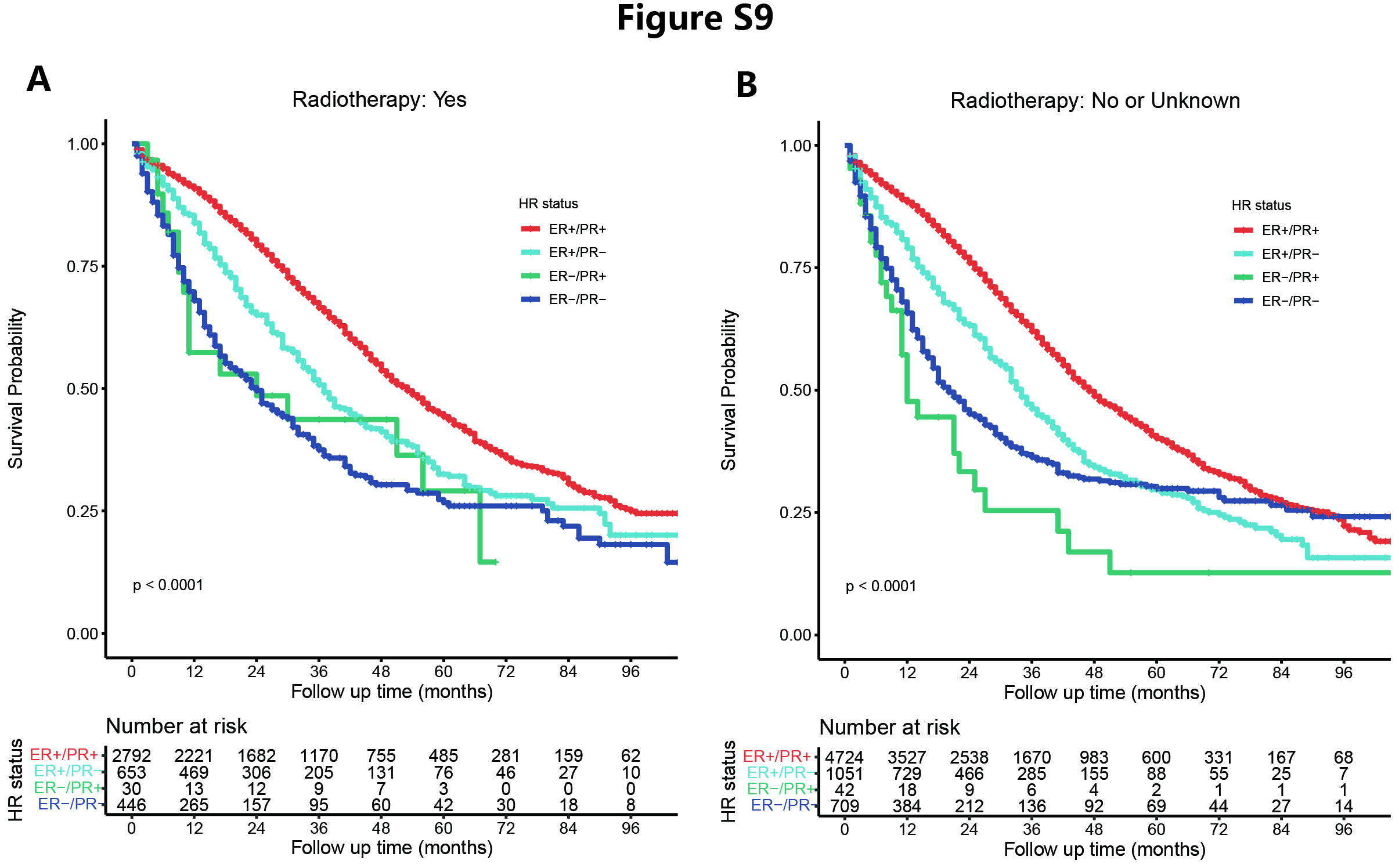

Supplement: Supplementary Figure 9 — Breast cancer-specific survival of bone metastatic breast cancer patients stratified by acceptance of radiotherapy, (A) accept radiotherapy, (B) no radiotherapy. [file Image_9.jpeg]
